# Methodological Considerations in Longitudinal Analyses of Microbiome Data: A Comprehensive Review

**DOI:** 10.3390/genes15010051

**Published:** 2023-12-28

**Authors:** Ruiqi Lyu, Yixiang Qu, Kimon Divaris, Di Wu

**Affiliations:** 1 Computational Biology Department, Carnegie Mellon University, Pittsburgh, PA 15213, USA; ruiqil@andrew.cmu.edu; 2 Department of Biostatistics, Gillings School of Global Public Health, University of North Carolina at Chapel Hill, Chapel Hill, NC 27599, USA; yqu@unc.edu; 3 Division of Pediatric and Public Health, Adams School of Dentistry, University of North Carolina at Chapel Hill, Chapel Hill, NC 27599, USA; kimon_divaris@unc.edu; 4 Department of Epidemiology, Gillings School of Global Public Health, University of North Carolina at Chapel Hill, Chapel Hill, NC 27599, USA; 5 Division of Oral and Craniofacial Health Sciences, Adams School of Dentistry, University of North Carolina at Chapel Hill, Chapel Hill, NC 27599, USA; 6 Lineberger Comprehensive Cancer Center, University of North Carolina at Chapel Hill, Chapel Hill, NC 27599, USA

**Keywords:** review, microbiome data, longitudinal analysis, statistical methods, deep learning

## Abstract

Biological processes underlying health and disease are inherently dynamic and are best understood when characterized in a time-informed manner. In this comprehensive review, we discuss challenges inherent in time-series microbiome data analyses and compare available approaches and methods to overcome them. Appropriate handling of longitudinal microbiome data can shed light on important roles, functions, patterns, and potential interactions between large numbers of microbial taxa or genes in the context of health, disease, or interventions. We present a comprehensive review and comparison of existing microbiome time-series analysis methods, for both preprocessing and downstream analyses, including differential analysis, clustering, network inference, and trait classification. We posit that the careful selection and appropriate utilization of computational tools for longitudinal microbiome analyses can help advance our understanding of the dynamic host–microbiome relationships that underlie health-maintaining homeostases, progressions to disease-promoting dysbioses, as well as phases of physiologic development like those encountered in childhood.

## 1. Motivation

The human microbiome, including metagenomics and metatranscriptomics, has recently taken a prominent role in our understanding of health and disease. Considerable resources and efforts are now being invested in studies measuring aspects of the microbiome, including multi-omics and longitudinal study designs. New insights have been gained by longitudinal microbiome data studies in human development [1], disease progression [2,3,4], medical treatment effects [5], and mortality [6]. Cross-sectional microbiome studies have been successful in uncovering novel key species with important roles in common human diseases [7]. Such findings provide motivation for additional microbiome studies and deeper analyses involving longitudinal study designs, because microbiomes are naturally dynamic, sensitive to disease progression, and change across the lifespan. Longitudinal microbiome studies also allow for a better understanding of interactions among microbial community members, as well as interactions between microbial species/genes and the human host over time [8]. Microbiome data collected at multiple time points, often with time-matched and tissue-matched metabolomics data from the same participant, enable assessments of microbial trajectories, and the identification of important microbial biomarkers that could facilitate disease prediction or inform disease management [9].

**Figure 1:**
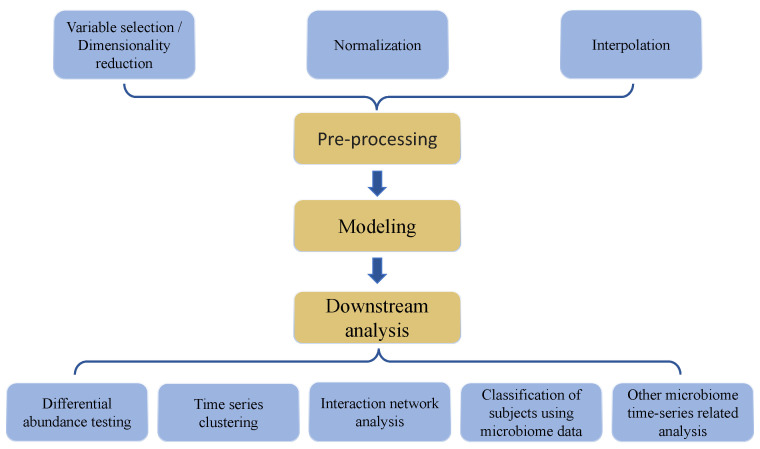
Key elements and steps in data analysis process followed in most investigations involving microbiome time series data structures.

Longitudinal microbiome data analysis remains challenging because it not only inherits difficulties encountered in cross-sectional microbiome data, but also requires proper handling of correlation structures emanating from repeated sample collection of the same participants over time. Microbiome data are typically zero-inflated, over-dispersed, high-dimensional, and with complicated correlation structures [10,11,12]. Microbiome data can also be compositional or relative, with sample-level summation to one or a large constant [13]. First, for both cross-sectional and longitudinal microbiome data, common analysis steps (Figure 1) include pre-processing (e.g., scaling, normalization, and batch effect correction), model fitting, and downstream analysis (e.g., differential abundance analysis, clustering, and classification) [14]. Importantly, longitudinal microbiome data may have specific needs in these analysis methods, according to time-specific hypotheses and assumptions. Second, specifically for longitudinal data, real-world scenarios of data collection at multiple time points in a sizeable cohort (for example, >30 subjects) often include irregularities of time intervals and missingness, as well as abrupt state transitions [15]. One proposed solution to these longitudinal study-specific challenges is the employment of deep-learning-based interpolation during preprocessing [16].

The importance of overall study design in cohort studies cannot be overemphasized, as the quality of evidence and downstream inferences hinge on a rigorous design [17]. When microbiome data are part of cohort studies, additional challenges exist. The analysis of time-series microbiome data requires careful consideration of issues ranging from preprocessing to parametric modeling and downstream analyses, necessitating the development and application of novel, suitable methods. The emergence of new questions in this research area has stimulated the need to conduct a thorough review to suggest more appropriate analysis methods for these complex data structures and, importantly, identify potential directions where more research and methods development are warranted.

In this paper, we sought to comprehensively review nuances in microbiome time series data that present analytical challenges, including preprocessing, modeling, and downstream data analyses (Figure 1). We review the currently available related computational approaches and methods. Specifically, in data preprocessing, we review approaches including normalization, variable identification, dimensionality reduction, and interpolation. In downstream analyses, we review differential abundance testing, time series clustering, host trait delineation, and inference of interaction networks.

## 2. Methods That Account for General Distribution Characteristics of Microbiome Data

Microbiome datasets are characterized by their compositional, non-negative, zero-inflated, over-dispersed, and high-dimensional nature, as highlighted in a recent review [12]. This intrinsic complexity elevates the difficulty of data analyses. Though many available methods exist to deal with these challenges in cross-sectional studies, we still include them briefly in this review because these issues are exacerbated when analyzing longitudinal microbiome data. The temporal dimension introduces additional layers of complexity, such as the need to account for time-dependent changes and inter-individual variability, making the interpretation and analysis even more intricate and demanding. Where extensions to longitudinal microbiome data exist, we discuss them after introducing the general methods.

### 2.1. Non-Negative Counts and Compositional Data

Most notably, output reads of sequencing data or assigned taxa generated by high-throughput sequencing (HTS) may not be informative themselves. In many current approaches, it is common to normalize counts by setting the sum of all operational taxonomic unit (OTU) components to be a constant value [13]. The challenge is compounded when these counts vary significantly over time, a feature often overlooked in cross-sectional studies. Weiss et al. [10] discuss normalization strategies, but longitudinal studies necessitate additional considerations for temporal fluctuations, as the relative abundance’s trend is not equivalent to the real abundance’s trend. In longitudinal studies, this compositional nature needs to be interpreted with caution, especially when considering the time-dependent changes in microbiome composition [11].

More recentl approach allows for information to be retained in the relative proportions (e.g., ratios) between the members of the microbial community, a feature that leads to intrinsic inner-correlation of taxa and has severe implications for assumptions that can be made for the data and the implementation of analytical methods. As a result, traditional methods can produce biased estimates when applied to microbiome data. For example, none of the three distance or dissimilarity matrix-estimating methods that dominate the relevant literature, UniFrac (both the weighted and unweighted variants), Bray–Curtis, and Jensen–Shannon divergence, account for the compositional nature of these data [18]. To overcome this issue, Aitchison [19] proposed that the underlying relationships between taxa can be captured by treating the data as ratios and proposed the centered log-ratio (CLR) transformation, which can be performed before computing the distances to mitigate the challenges imposed by the compositional nature of the data. A factor that may be easily ignored is that microbiome data are naturally non-negative. This can also be a problem for statistical inference [20] because the direct application of Gaussian-based traditional methods may be inappropriate.

### 2.2. Zero-Inflation

Another important and well-recognized feature of microbiome data is their zero-inflation, which refers to the presence of a higher proportion of zeros compared to what would be expected under a typical distribution, Poisson or negative binomial, ranging between 70–90 percent [21]. Zero-inflation poses specific challenges in longitudinal settings, where the pattern of zeros can vary over time. There are many different kinds of zeros in microbiome data, including sampling zeros (unobserved due to limited sample size or below detection), rounded zeros (not actually zeros), and structural zeros (true zeros). These zeros hamper efforts to determine whether the data provide “evidence of absence” (i.e., the true absence of a particular taxon in a sample) or “absence of evidence” (i.e., the inability to detect a taxon due to sampling or measurement limitations). Estimates of conventional parametric models are not trustworthy for samples that consist mostly of zeros [22]. The excess zeros present in the taxonomic abundance reduce the power for generating inferences regarding low-abundant taxa by using either standard parametric models [23] or non-parametric methods [24]. Several zero-inflated models have been proposed to help overcome the data sparsity issue, including zero-inflated Beta regression with random effects (ZIBR) [25], negative binomial and zero-inflated mixed models (NBZIMM) [26], and fast zero-inflated negative binomial mixed model (FZINBMM) [27].

### 2.3. Overdispersion

Overdispersion is another common problem encountered in microbiome data analysis, referring to the occurrence of greater variability in the data than expected under standard statistical models, often necessitating specialized analytical approaches for accurate interpretation. This variability is often more pronounced in a temporal context, with overdispersion potentially varying at different time points. Overdispersion can be caused by many factors, including the presence of a large number of zero-inflated or rare taxa, differences in sampling depth between samples, and variations in the underlying biology of the microbial communities under study. Consequently, while Poisson models are commonly used for count data, their assumption of an equal mean and variance reduces their applicability to the traditionally over-dispersed microbiome data [28]. Instead, negative binomial models with an additional dispersion parameter are more popular and better-suited to account for the over-dispersion [29].

Mixed models, like NBZIMM [26] and FZINBMM [27], offer flexible frameworks to account for overdispersion by incorporating additional variance components, such as dispersion parameters or random effects. The dispersion parameter serves as a scaling factor for the variance and is aimed at capturing the extra variability that is not explained by the mean of the distribution [29]. By allowing this parameter to vary, a mixed model can adjust its variance structure, and thus better accommodate the true underlying data variability. Random effects can provide another means to account for overdispersion. By including random effects that capture subject-specific variability, the model can better account for the extra variation observed between participants, often arising from missing covariates or unmeasured variables. These random effects can represent unobserved heterogeneity between study participants, or temporal correlations within participants, introducing an additional layer of complexity that can align the model closer to the underlying data-generating process. The random effects play a dual role: they account for the inherent correlations within grouped data [26] and simultaneously provide an additional layer of variance, mitigating overdispersion [29]. Besides handling overdispersion, mixed models benefit from being flexible in their specification of variance–covariance structures. This adaptive nature ensures that the model is not restricted by stringent assumptions and can evolve as required.

### 2.4. High Dimensionality

Most microbiome datasets are inherently high-dimensional; the number of taxa can easily be in the hundreds or thousands [12], while the sample size remains relatively modest. This high dimensionality presents unique challenges in longitudinal studies, because time introduces an additional dimension with a more complicated correlation structure. Genomics applications (i.e., metagenomics) can push the dimensionality to an extremely large scale wherein data dimensions can grow exponentially with the sample size—a phenomenon termed ultrahigh-dimensional data [30]. For example, making inferences regarding interactions or connectivity between taxa or their genes often is an NP-hard (non-deterministic polynomial-time hard) problem [31], rendering even heuristic or greedy methods impractical, unless preliminary dimensionality reduction and pruning occur. In addition, microbial features (e.g., genes) may be correlated due to their relatedness (i.e., genetic relationship) in the phylogenetic tree [32].

## 3. Challenges in Longitudinal Analysis of Microbiome Data

### 3.1. Challenges in Temporal Study Design and Sample Collection

In addition to the microbiome-specific data distribution characteristics mentioned above, microbiome time series analyses present unique challenges that arise from the temporal data collection in the study design. This section specifically focuses on the dynamics and complexities unique to longitudinal microbiome data, setting it apart from general microbiome studies as discussed in other literature [11,12]. Most microbiome insights have emerged from cross-sectional studies [33], which provide only a single time point snapshot [34]. Naturally, the absence of a temporal dimension limits one’s ability to make inferences regarding health and disease-informative microbial changes and underscores the urgent need for such time series data to be collected and analyzed. Additionally, the aggregation of microbiome data from different periods or conditions (e.g., “batches,” “cohorts,” or “sampling groups”) often introduces substantial heterogeneity, introducing biases and confounders that complicate the differentiation between genuine biological signals and data collection or processing artifacts.

Recent time-series studies reveal a substantial dynamism of the microbiota of humans and animals, underscoring the importance of such longitudinal microbiome data. Though large-scale studies such as the human microbiome and MetaHIT projects explored the phylogenetic and functional composition of the healthy human microbiota and its inter-individual variation [35,36], time-series data on gut microbiomes, especially datasets including samples from many different participants, remain rare due to the challenging and expensive data collection process [34]. Dense time series and covariates that explain gut microbiome dynamics, such as diet and social interactions, are also difficult to collect. As a result, most datasets either include many time points from a few participants, or relatively few time points from larger numbers of participants, limiting their statistical power [37,38,39], and the discrepancies can even lead to seemingly contradictory results [40].

Aspects and characteristics of microbiome collection and measurement can influence the quality of the obtained data, introducing variation between different data batches. Published protocols detailing clinical aspects of microbiome sample collection, as well as specimen handling, storage, nucleic acid extraction, sequencing, QA/QC, pre-processing, and ultimately data analysis and inference, are rare but do exist [41]. Sequencing techniques, variation in spatial aspects and temporal frequency of sampling, as well as the availability of replicates, are all features that can strongly influence the results of a time series analysis. While frequent sampling is crucial to capture the richness of microbial communities’ variability [42], the ideal sampling frequency depends on study-specific characteristics, including the expected variability and study hypotheses [2,3,4,43]. Current methods used to obtain estimates of microbial abundance are still noisy and, when multiple technologies are combined (i.e., next-generation sequencing and qPCR), the resulting measurement error models become complex [20] and can greatly affect downstream analyses. In general, dynamic environments increase sample heterogeneity, but unlike other biomedical domains where almost continuous temporal sampling is feasible, this is not currently possible for most human microbial niches including the gut [44].

Most currently available microbiome time series datasets cover short time ranges and/or have gapped time points [15] due to practical limitations in sample collection and processing, although, ideally, data collection should regularly and repeatedly collect and analyze samples from the same individuals over long periods of time [45]. Unsurprisingly, irregular microbiome sampling can pose challenges for investigators, as it may not conform to standard time series models. Meanwhile, many methods for time series clustering, classification, and regression work better with regularly-sampled data as inputs, creating a need to appropriately handle (i.e., transform) the irregularly collected samples [16].

### 3.2. Challenges in Appropriate Handling of Longitudinal Features in Microbiome Data

Longitudinal studies present both opportunities and challenges in microbiome research. The temporal dimension adds complexity but also offers richer insights into dynamic processes, patterns, and interactions within microbial communities compared to what cross-sectional studies can offer. The process of collecting samples over an extended period magnifies the nuances in community composition. These changes may be attributed to a multitude of factors, including variations in host environment, aging, behavior, or diet changes, medications, or the activity of other species. This complex interplay makes it difficult to disentangle the individual effects of each feature and decipher their cumulative impact on the overall microbial community. Additionally, incorporating time-related experimental parameters like the frequency and timing of sample collection, as well as the tracking of host life cycle events and environmental changes, is crucial. This approach aids in interpreting temporal changes and identifying long-term microbial patterns.

In time series studies of microbiome data, some of the aforementioned problems are more pronounced. The microbial communities’ composition is often presented in relative proportions, so an increase in one taxon’s abundance can misleadingly appear as a decline in others, due to their relative nature. Such relativistic interdependence can lead to the misinterpretation of spurious interactions and dependencies, obscuring the true temporal dynamics of the microbial community. To address this, detailed metadata collection, including clinical and lifestyle factors, alongside microbiome samples, becomes essential. Longitudinal studies also introduce serial correlations, wherein consecutive observations are correlated with each other, a feature that must be considered and accounted for in time series data analysis [46].

Addressing these challenges necessitates a robust methodological framework. Mixed-effects models [25,26,27] and state space models [47,48,49] have emerged as promising tools for handling the complexities of longitudinal microbiome data. Moreover, specialized time-series clustering techniques and network-based approaches may be employed to discover latent patterns and relationships within the microbiome data [42]. These tools partition the data based on their similarity over time, and this way facilitate the identification of trends and interactions within the microbial community while handling the compositional and sequential nature of those data.

## 4. Methods for Preprocessing

**Figure 2:**
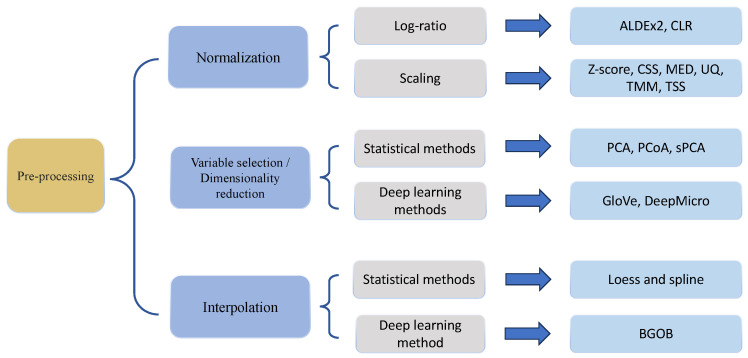
Overview of methods for preprocessing of microbiome data.

As with the other count-based sequencing data, appropriate preprocessing steps, summarized in Figure 2, to deal with library size effects and characteristics of data distribution as well as study design, are necessary prior to microbiome data analysis in both cross-sectional studies and longitudinal studies. On one hand, there exist comprehensive reviews and research papers that elaborate on normalization, preprocessing, and model selection [10,11,50], though mostly for cross-sectional studies. Therefore, in this section, we will only briefly discuss preprocessing steps commonly used in microbiome analysis including normalization, variable selection, and dimensionality reduction. On the other hand, specifically for longitudinal microbiome data analysis, we will focus on discussing the interpolation of missingness in terms of data collection time points/intervals. Although there are very few methods to address the interpolation problem in longitudinal microbiome data, it is a very important future direction to improve upon, that will affect the results of the downstream analysis.

### 4.1. Normalization

Normalization is critical for microbiome data preprocessing, not only for time series normalization but also for ensuring the comparability and accuracy of diverse data sources [10]. Motivated by an earlier approach to handling compositional data [19], several log-ratio-based methods have been proposed by converting the observed abundances to log-ratios within each sample. Then, by considering the log-ratios of all taxa with respect to a common reference taxon or a suitable function of all taxa, these methods explicitly eliminate the effect of the sampling fraction [51]. For example, ALDEx2 [52] uses a pre-specified taxon as a reference and transforms the observed abundances of each taxon to log-ratios relative to the reference. CLR normalization [53] is one of the most popular methods for microbiome data analysis. Instead of using a pre-specified taxon as a reference as in ALDEx2, CLR uses the center of mass of all taxa as the reference. To this end, it subtracts the logarithm of the mean abundance of all taxa from the logarithm of the abundance of each individual taxon. The mathematical expression for CLR normalization is shown in Table 1, where *L* is the total number of taxa, $Y_{l}$ is the abundance of the *l*-th taxon. CLR is implemented in the “LinDA” method [54], wherein a linear regression model is fit and then used for hypothesis testing using the centered log-ratio transformed data.

Normalization through scaling is another widely employed approach. This method involves dividing the taxonomic abundances within a feature table by a designated scaling or normalization factor. As such, normalization via scaling effectively mitigates biases arising from dissimilar sampling fractions, ensuring a more equitable representation and comparison of microbial abundances [10]. However, the scaling-based methods discussed here do not inherently account for the compositional nature of microbiome data.

Z-score transformation, also known as standard score transformation, is yet another statistical method that can be used to normalize microbiome data. This transformation is achieved by applying the formula shown in Table 1. $Y_{l}$ is the abundance of the *l*-th taxon, $\mu_{Y}$ is the mean of the population, and $\sigma_{Y}$ is the standard deviation of the population. This transformation can make the data more amenable to techniques that rely on Gaussian distribution assumptions. In the context of microbiome data, applying the Z-score transformation can help mitigate issues related to heteroskedasticity and non-normality.

Many other methods exist and can be used for scaling-based normalization. The median (MED) normalization method computes the median intensity of features within a sample and scales data by dividing individual intensities by this median value, offering robustness against outliers. Similarly, the upper quartile (UQ) method focuses on the 75th percentile intensity to account for range variations among samples. Trimmed mean of M-values (TMM) normalization [55] calculates gene expression ratios between samples, excluding extreme ratios to compute a scaled mean. Lastly, total-sum scaling (TSS) adjusts the sum of feature intensities to a fixed value, thereby standardizing total intensities across samples. ‘T’ in TSS is a fixed total sum value to which all samples’ total intensities are standardized. The choice of ‘T’ could be based on a typical or median value observed in the dataset or a predetermined standard that aligns with the research objective. Cumulative-sum scaling (CSS) [56] optimized TSS by iteratively scaling data to maintain a consistent cumulative sum (C), typically determined based on dataset characteristics, like some percentile of each sample’s nonzero count distribution, effectively addressing differences in signal intensities across samples.

Rarefaction is a rigorous way to address the existence of samples’ different library sizes, by assuming that similar sequencing reads are expected across samples. Nevertheless, this approach may be limited by low power if the study includes samples with much lower sequencing depths than others [57].

### 4.2. Variable Selection and Dimensionality Reduction

Variable selection and dimensionality reduction can play an important role in microbiome data analysis preprocessing, given the inherent complexity of most microbiome datasets that contain large numbers of features (e.g., taxa, genes, gene families). These techniques seek to retain the most essential information from the data, by identifying key taxa and representing the initially high-dimensional data in a more parsimonious manner. Here, we discuss a generalized context of variable selection and dimensionality reduction, as time-series-specific methods are underdeveloped, even if, in practice, some investigators may treat longitudinal variables as common variables. We will point out challenges in this domain as it pertains to time series data.

Variable selection methods typically focus on isolating the most significant microbial taxa or features while excluding those with low relevance or no information content. This often involves the removal of taxa exhibiting low prevalence or abundance, where prevalence denotes the proportion of samples detecting an taxa, and abundance refers to the relative quantity of a taxon within a sample. Among all variable selection techniques, LASSO (least absolute shrinkage and selection operator) [58], and other other LASSO-like penalized regression methods (including group LASSO [59] and sparse group LASSO [60], etc.) are most popular. These methods effectively shrink coefficients of less important features to zero, thereby selecting only the most correlated taxa or features. In microbiome datasets, they help in identifying taxa that have significant effects while preventing overfitting in models with a large number of predictors [61].

Complementing variable selection, dimensionality reduction seeks to represent the initially high-dimensional data with lower-dimension vectors. While traditional methods like principal component analysis (PCA) are commonly used, their effectiveness is frequently limited in microbiome data applications. PCA requires normalization due to its sensitivity to variable scales to avoid distortion in results by variables with larger ranges. Modifications like principal coordinates analysis (PCoA) [62] and sparse principal component analysis (sPCA) [12,63] offer more flexibility. The PCoA method focuses on a distance matrix between samples rather than the sample covariance matrix. Instead of using the original observed data, PCoA decomposes the distance matrix, leveraging the statistical properties inherent in these distances, which affords PCoA greater flexibility than PCA. Alternatively, sPCA has been employed to cluster taxonomic profiles with similar temporal patterns [12,63]. In the first step of sPCA, the original data matrix is summarized using linear mixed model splines (LMMS), resulting in a reduced matrix. In the second step, PCA further reduces the dimensions, identifying strongly correlated profiles via their loading coefficients. The optimal number of principal components is determined using statistical measures like the average silhouette coefficient. Compared with PCA, sPCA focuses on subsets of taxonomic profiles that are highly correlated within a component, emphasizing those that conform to the average cluster profile versus the outliers.

Recently, methods that borrow ideas from natural language processing (NLP), such as word embedding techniques, have been developed for microbiome data preprocessing. For example, Tataru and David [64] used the GloVe embedding algorithm [65] from NLP for data preprocessing prior to using a random forest approach to predict a binary outcome. In that study, the dataset preprocessed by GloVe outperformed the one preprocessed by PCA.

Deep learning methods are strongly emerging in the field of microbiome variable selection. These models can abstract microbiome data with large numbers of taxa into low-dimensional vectors while retaining the maximum information possible. For example, DeepMicro, proposed by Oh and Zhang [66], relies on a deep learning-based approach to reduce the dimensionality of microbiome data. The method employs an autoencoder, i.e., a neural network that reconstructs its input *x* for the dimensionality reduction. DeepMicro comprises an encoder function $f_{\phi }\left(\cdot \right)$ and a decoder function $f_{\theta }^{\prime }\left(\cdot \right)$ , with encoder parameters ϕ and decoder parameters θ , respectively. By minimizing the reconstruction loss between an input *x* and a reconstructed input $x^{\prime }$ , as shown in Equation (1), the best parameters for ϕ can be identified. After the best $f_{\phi }\left(\cdot \right)$ is found, it can be applied to individual samples to significantly reduce the data dimensionality.

(1) $$L\left(x,x^{\prime }\right)={∥x-x^{\prime }∥}^{2}={∥x-{f^{\prime }}_{\theta }\left(f_{\phi }\left(x\right)\right)∥}^{2}.$$

Evidently, more computational methods need to be developed for dimensionality reduction in longitudinal microbiome data. One motivation could be to answer whether the same features should be retained as selected across multiple times, and how this can be achieved using the discussed tools. The selection of variable selection and dimensionality reduction methods is greatly influenced by the specific objectives of the downstream analysis. For instance, in scenarios involving time-series prediction or host trait regression, coupled with the selection of predictive species, LASSO-like methods are appropriate. Moreover, for tasks focused on deriving embeddings or low-dimensional representations of samples or features, methods like PCA, PCoA, and sPCA are more suitable. Lastly, in cases where the sample size is ample and interpretability is not a primary concern, exploring deep learning-based methods can be highly beneficial.

### 4.3. Interpolation Dealing Irregular Longitudinal Data

In large time series cohort studies, individual participants may not contribute microbiome samples exactly at the study-designated time points and/or may not have equal time intervals across subjects, even in well-designed studies [2,3]. To demonstrate the challenges, we give one example. In the study of longitudinal microbiome data of Early Childhood Caries (ECC), the oral microbiome data from salivary samples were collected at roughly six time points from birth, from 2 months to 48 months, to provide an ordered temporal trajectory of the oral microbiome development as children grew and developed their natural dentition [4]. The standard deviation of collection time is between 1 and 3 months and there are missing time points for some subjects. Although the standard deviation is not large compared to the duration of the study, changes in the oral microbiome can happen in the early months. For increased testing power and full leverage of the data, it is best to deal with such irregular sampling caused by the above-mentioned variation of collection time points. This is a common real-world issue, that can create significant challenges for several downstream data analyses, including the temporal trend-based clustering and classification, and the inference of interaction networks across microbial features. Here, interpolation can assist by formulating models that accurately depict the temporal data dynamics and by offering data smoothing and noise reduction.

Loess (or lowess) and spline are two commonly-used interpolation methods that can be used for longitudinal microbiome data analyses. ‘Loess’ stands for “locally estimated scatterplot smoothing”, and is used interchangeably with ‘lowess’, or “locally weighted scatterplot smoothing”, a non-parametric method based on fitting a series of local linear regression models. The weight assigned to each data point diminishes as its distance from the point of interest increases and, this way, points closer to the one of interest are given upweighted and those further away are downweighted. The loess method is particularly effective in analyzing data with underlying non-linear trends, providing a smooth curve that represents the underlying data pattern. In contrast, a spline is a piece-wise continuous polynomial function designed to approximate a curve. This curve is constructed from a series of polynomial segments that are seamlessly connected at specific points, known as “knots”, creating a smooth curve that encompasses all data points.

Several interpolation methods have been employed for microbiome time series data analyses. For example, Shields-Cutler et al. [67] introduced splinectomeR, an R package that employs loess for data summarization, facilitating hypothesis testing in longitudinal studies. This is particularly relevant for omics data that exhibit non-linear trends. To illustrate the importance of spline-based methods, Luo et al. [68] introduced metaDprof, a tool that leverages splines to estimate time trends. Specifically, metaDprof applies a smooth spline function across all samples to capture the underlying time trends. The processed data are then adeptly utilized for downstream analyses that include feature selection and differential abundance testing.

In the rapidly evolving field of microbiome research, deep learning methods have shown promising advantages, particularly with large sample sizes. Qu et al. introduced the Bidirectional GRU-ODE-Bayes model (BGOB) [16], an innovative approach that leverages a modified neural ODE [69] for enhanced microbiome data interpolation. The BGOB model demonstrates superior performance over traditional spline-based methods in high-dimensional contexts based on their simulations. Further, the simulations also reveal that datasets interpolated using BGOB yield improved outcomes in clustering tasks. This advancement is particularly relevant as longitudinal microbiome datasets continue to grow, underscoring the potential of deep learning in handling large-scale data effectively.

## 5. Statistical Models Suitable for Longitudinal Microbiome Data

In this section, we present several parametric models commonly employed in longitudinal microbiome analyses. These models make different assumptions about the data but are all useful for extracting parameters of interest, enabling subsequent statistical testing and inferences. To help readers appreciate these models’ utility in longitudinal microbiome research, we elaborate on their different assumptions and underlying logic. A brief summary of these models is presented in Table 2.

### 5.1. Mixed Effect Models

In microbiome time series analyses, mixed effect models are popular for capturing both population-level trends (fixed effects) and individual variations (random effects). Linear mixed models, for instance, are predicated on the assumption that random effects are independent, the parameters exhibit linearity, and the errors follow a normal distribution. Mixed effect models are particularly adept at handling correlated data, a common occurrence in longitudinal studies.

Linear mixed effect models are commonly used in microbiome longitudinal analysis [77]. For instance, Bokulich et al. [70] employ linear mixed effect models, considering time, group, and gender as fixed-effect covariates while treating individual participants as a random effect. By doing so, these investigators were able to account for commonalities in the study population while still recognizing the unique microbial interactions within individuals and environments. Nevertheless, these models may not perform well in the presence of highly nonlinear relationships and may require careful specification of the fixed and random effects, somewhat limiting their flexibility [78].

Zero-inflated mixed effect models, including the zero-inflated beta regression model (ZIBR) proposed by Chen et al. [25], the negative binomial and zero-inflated mixed models (NBZIMM) proposed by Zhang et al. [26] and the fast zero-inflated negative binomial mixed model (FZINBMM) proposed by Zhang et al. [27], are commonly employed as they effectively incorporate the characteristic of zero-inflation of microbiome data. These models operate under the assumption that data are generated from a mixture of two different distributions, allowing them to manage both the presence/absence and non-zero abundance of taxa. By employing a two-part model that combines a point mass at zero with a continuous distribution for positive values, zero-inflated models provide a more nuanced representation of microbial abundance. However, these models can be challenged and misfit when the data contain too many or too few zero values. Additionally, zero-inflated models may not always handle compositional data effectively, as they are primarily designed to address zero-inflation rather than the relative relationships between members of a microbial community.

### 5.2. ARIMA Models

Autoregressive integrated moving average (ARIMA) models are also widely-used in time series analysis and are known for their ability to capture temporal trends. By combining autoregressive (AR) terms, differencing (I) to make the series stationary, and moving average (MA) terms, ARIMA models can effectively model the dependencies between observations. This multifaceted approach makes ARIMA models particularly suitable for microbiome time series analyses, wherein complex temporal relationships are expected. For example, Benjamin et al. [71] utilized ARIMA to analyze microbiome time trends and infer between-species interactions. The model’s ability to integrate temporal aspects allows for a more comprehensive characterization of dynamic changes encountered in human microbiomes. However, ARIMA models assume stationarity of the time series (or achieved through differencing), finite variance, and uncorrelated white noise errors, while these assumptions are often violated in practice. Moreover, ARIMA models may not handle seasonal patterns well without extensions, e.g., extension as the seasonal autoregressive integrated moving average (SARIMA), and can be sensitive to outliers [79].

### 5.3. State Space Models

State space models refer to a class of probabilistic graphical models that describe the probabilistic dependence between latent state variables and observed measurements [80]. These models are valuable in modeling and extracting hidden state patterns that might influence the observed data. Chen et al. [72] proposed using a high-dimensional linear state space model to study the dynamics of microbiome interactions, leveraging their ability to capture complex relationships. By employing this approach, these investigators were able to construct a dynamic microbial interaction network (MIN), considering both system and measurement noise. State space models’ ability to deal with high-dimensional data and preserve the sparsity property of MINs makes them powerful tools in the analysis of complex microbiome interactions. However, state space models may not perform well in the presence of nonlinear dynamics and can have high computational complexity for large state spaces, posing challenges for some applications.

### 5.4. Principal Trend Analysis Models

Principal trend analysis (PTA), originally proposed for analyzing time series genomics data [81], is a statistical method used to identify and assess the main trends in a dataset over time or across different conditions. The model assumes linearity of trends and homogeneity of variance across time points. PTA integrates latent factor models for dimensionality reduction with spline-based methods for temporal structure modeling, allowing investigators to observe the progression of variables and their interactions over time. PTA aids in deciphering complex data structures and temporal variations, extracting principal trends of time-course data, and facilitating predictions. Microbiome trend analysis (MTA) proposed by Wang et al. [73] is an extension of PTA in microbiome data analysis, by incorporating taxonomic information from the phylogenetic tree structure. This adaptation allows for the identification of dominant contributions to principal trends within the microbiome, recognizing both temporal patterns and phylogenetic relationships. The MTA model represents a significant advancement in the field, bridging the gap between genomics and microbiome analysis, and provides a robust framework for understanding the complex dynamics of microbial communities. While this approach allows for nuanced trend analyses, it may not capture complex nonlinear trends and can be sensitive to the choice of phylogenetic tree structure.

### 5.5. Generalized Lotka–Volterra Models

Generalized Lotka–Volterra (gLV) models are the most commonly used models in microbiome time series analyses.The basic gLV model can be constructed as follows. For *L* taxa measured in *S* participants, we denote the abundance of taxon *l* in participant *s* as $f_{ls}$ . Suppose there are total *P* perturbations, the rate of change of the abundance of taxon *l* in participant *s* is expressed as Equation (2) [74] in the gLV model.

(2) $$\frac{df_{ls}}{\;dt}=\alpha_{l}f_{ls}\left(t\right)+\sum_{j=1}^{L}\beta_{lj}f_{ls}\left(t\right)f_{js}\left(t\right)+\sum_{p=1}^{P}\gamma_{lp}f_{ls}\left(t\right)u_{p}\left(t\right).$$

The α parameters represent unbounded growth rates, the β parameters represent pairwise microbiome–microbiome interactions, and the γ parameters represent effects of the perturbations. Numerous techniques exist for the estimation of target parameters $\theta_{l}={\left[\alpha_{l},\beta_{l1},\cdots ,\beta_{lL},\gamma_{l1},\cdots ,\gamma_{lP}\right]}^{T}$ . For example, linear model methods including ridge regression and Bayesian algorithms can be used to understand the underlying dynamics of a given system. Bucci et al. [74] propose MDSINE, which uses one MLE method (maximum-likelihood constrained ridge regression) and two Bayesian methods (Bayesian adaptive lasso and Bayesian variable selection) to estimate the model parameters $\theta_{l}$ .

Stein et al. [75] use the gLV model to study microbial interactions, interactions between commensal and pathogenic bacteria, and the effect of antibiotics on the microbial community. Gibson et al. [20] modify the traditional generalized Lotka–Volterra (gLV) model by replacing piecewise interaction with interaction modules, allowing for the automatic discovery of clusters in the microbial community during the parameter inferring process. Additional methods exist that provide user-friendly interfaces for employing gLV models. For example, MetaMIS, proposed by Shaw et al. [31], constructed an easy-to-use graphical user interface (GUI) and Web-gLV proposed by Kuntal et al. [76] embeds the gLV model into a web browser.

### 5.6. Bayesian Models

Bayesian statistics plays an important role in microbiome time series analyses. While some Bayesian models might also be categorized under other sections, including the zero-inflated models and the state-space models, we have included them here due to their reliance on Bayesian methods for parameter inference. Äijö et al. propose TGP-CODA [48], which employs a Gaussian process model to ascertain the state space covariance matrix for longitudinal count data, taking into account the temporal correlation of consecutive time points. It also integrates a hierarchical model to tackle over-dispersion and introduces an independent parameter for technical zeros. MALLARD, which is proposed by Silverman et al. [47], is a member of the state space model family. It utilizes the inverse of the isometric log-ratio transform (ILR) to adjust parameters that follow a multivariate normal to a sum constraint of 1. Luminate, proposed by Joseph et al. [49] also uses a state space model to determine taxonomic relative abundance from longitudinal microbiome data, and differentiates between biological and technical zeros. The strength of Bayesian methods lies in their ability for parameter inference without the necessity to integrate all missing values. Additionally, Bayesian models can be highly flexible, which can be useful when dealing with the complex nature of longitudinal microbiome data. However, it is crucial that investigators accurately define their model priors, otherwise, Bayesian methods may under-perform.

## 6. Downstream Analysis of Longitudinal Microbiome Data

**Figure 3:**
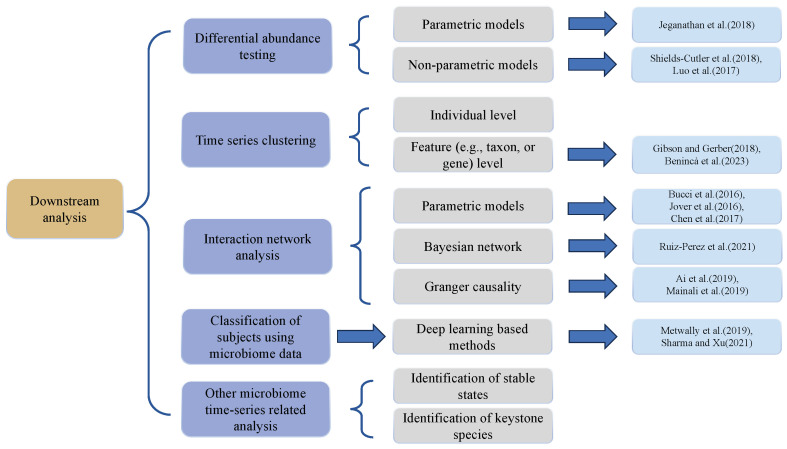
Overview of downstream analysis approaches for longitudinal microbiome data. (The listed references are: Jeganathan et al. (2018) [82], Shields-Cutler et al. (2018) [67], Luo et al. (2017) [68], Gibson and Gerber (2018) [20], Benincà et al. (2023) [83], Bucci et al. (2016) [74], Jover et al. (2016) [84], Chen et al. (2017) [72], Ruiz-Perez et al. (2021) [85], Ai et al. (2019) [86], Mainali et al. (2019) [87], Metwally et al. (2019) [88], Sharma and Xu (2021) [89]).

In this section, we will present and discuss several comprehensive analysis tasks and corresponding methodologies as they apply to longitudinal microbiome data analyses. These include differential abundance testing [21], time series clustering, interaction network analyses, host trait classification, and other relevant microbiome time-series analyses. The outline of this section is shown in Figure 3. Differential abundance testing compares relative abundances of various microbial taxa across different time points or conditions. Time series clustering categorizes microbial community samples by the similarities in their temporal dynamics, facilitating the recognition of analogous patterns and trends within the data. Interaction network analysis formulates networks of microbial taxa derived from their distribution patterns, aiding in the identification of keystone taxa and potential catalysts of change within the microbial community. The classification of participants, e.g., hosts, predicts host traits such as disease groups and treatment responses based on the temporal dynamics of the microbial communities under study.

One critical point related to both the study design and the downstream analysis is how to choose the right downstream methods and how to understand the downstream analysis results based on the study design including study cohorts and the specific clinical quotations to be answered. These following two examples of longitudinal microbiome studies represent two types of studies. First, the longitudinal ECC study started from the similar time point of birth to collect oral microbiome data until age 5, with 134 subjects and a maximum of six time points [4]. Such studies have clear starting points like birth, disease onset, or treatment, so that the time trajectory of the microbiome at the populations level is meaningful so that the complicated trajectory related questions and computational methods are suitable. On the other hand, another type of study may have no clear starting points. For example, the longitudinal microbiome data collected from the adult Inflammatory bowel disease (IBD) has followed 132 subjects for one year during disease (up to 24 time points each) [2]. Given that subjects in this cohort are adults with pre-existing IBD undergoing potentially varied treatments, the time trajectory of their microbiome at the population level becomes complex to discern, suggesting that employing mixed models, which account for repeated measurements, can be an effective analytical strategy.

### 6.1. Temporal Differential Abundance Testing

Differential abundance (DA) testing in the context of longitudinal microbiome data analysis is typically employed to identify differences in the abundance of microorganisms across time and between subjects. The DA analysis that can be performed using statistical models in Section 5 can also seek to detect whether taxa exhibit significantly different behaviors in different subjects and to pinpoint the time intervals during which these differences develop or manifest. A variety of methodologies and tools have been developed to address these complex questions, each with unique features and assumptions.

Some of the applicable methods employ parametric models, as discussed previously. There are also nonparametric methods, such as the block bootstrap method (BBM) proposed by Jeganathan et al. [82]. BBM employs a “moving block bootstrap” technique that constructs and resamples blocks of temporally-related observations with replacement. It is important to note that this technique accounts for autocorrelation, unequal library sizes, and within-subject data dependencies, without relying on a specific data distribution. By utilizing an overlapping block approach and modified empirical sub-sampling to optimize block size, BBM has been shown to minimize mean squared error for most statistics. However, this approach requires significant computational resources and an ample number of time points, with at least five needed to specify two critical tuning parameters. Despite its flexibility and strengths, such as having a high true positive rate, the method still faces challenges with sparsity and variability. It does not account for other variables, and its effectiveness hinges on having an equal number of observations across subjects. The method’s primary focus lies in the identification of differences in microbial abundance between sample groups without quantifying these differences.

Unlike other approaches, SplinectomeR, which is proposed by Shields-Cutler et al. [67], does not rely on a pre-specified model for differential abundance testing. Instead, it employs two primary functions, the permuspliner and the sliding spliner functions. The permuspliner function determines whether two groups of individuals show significantly different trajectories over time, while the sliding spliner function tests the data series at defined time intervals. Both these functions are grounded in permutation testing, deriving *p*-values post data preprocessing via splines. The SplinectomeR package leverages weighted local polynomials (loess splines) for summarizing and testing longitudinal data. Meanwhile, the model may be sensitive to outliers and cannot handle compositional data.

Another approach developed for temporal differential abundance testing is metaDprof proposed by Luo et al. [68]. These investigators employ the metaDprof method by first using smoothing spline models to fit the time trends under the null hypothesis (one curve fitting) and under the alternative hypothesis (two curves fitting). A goodness-of-fit test statistic is then computed for the two models separately, and an F-statistic is computed based on them. Subsequently, permutation-based methods are used to calculate the *p*-value of the observed F-statistic and to determine whether the two curves are differentially abundant. After identifying features with significant differential abundance, in their paper, the investigators employ permutation-based methods for time interval detection, wherein they calculate the ratio of relative change between the two areas under the curve for each unit interval and obtain *p*-values for the observed ratios.

### 6.2. Time Series Clustering

In microbiome data analyses, unsupervised clustering is often used to identify naturally occurring clusters, which can then be assessed for associations with characteristics of interest [90]. Various algorithms have been developed to perform clustering, including hierarchical methods like agglomerative clustering and k-medoids, topological methods such as self-organizing maps [91], and density-based methods like the DBSCAN [92] algorithm. These methods have been widely applied across different domains to identify informative patterns. The choice of optimization metrics is crucial in clustering, with the sum of squared error (SSE) being commonly used. Techniques to identify the optimal numbers of clusters include the “elbow” method and indices like the Calinski–Harabasz index or Silhouette width [50,93]. Hierarchical clustering methods, including the partitioning around medoids (PAM) [94] algorithm and hierarchical agglomerative clustering, are specialized techniques aimed at dividing data into groups that become increasingly similar at each level of hierarchy. The PAM algorithm is particularly favored due its robustness to noise and outliers, as it minimizes the sum of dissimilarities between objects and chosen representative objects (medoids), rather than using mean values, which can be sensitive to extreme values [95]. On the other hand, hierarchical agglomerative clustering takes a bottom-up approach, beginning with each data point as a single cluster and progressively merging them based on a chosen similarity measure, forming a dendrogram that visually represents the hierarchy of clusters. This process continues until all points are merged into a single cluster, allowing for various levels of granularity in clustering. For both the PAM and the agglomerative clustering methods, data normalization techniques are essential for ensuring comparability and accuracy in clustering.

For time series analyses, clustering methods are adapted to handle temporal patterns and sequences. Classic time series clustering methods have been summarized by Liao [96], focusing on recognizing patterns that evolve over time. Generally, most clustering approaches rely on distance or correlation metrics [97], assuming that time points from different participants align perfectly. Yet, in practice and in many longitudinal studies, there is no assurance of perfectly consistent sampling times across the entire study population, making interpolation a necessary step in preprocessing. However, some methods have been developed to relax the stringent requirements for pairwise time point matching. For example, dynamic time warping (DTW) has been introduced as an alternative to the sum of squared differences, offering a more sophisticated approach by recognizing temporal patterns that may be out of phase or misaligned in time. DTW allows for more accurate alignments of observations by stretching or compressing parts of the time series, considering various paths through a cost matrix and using cumulative distance calculations to determine optimal paths.

Clustering can be performed at either the participant level or the feature (e.g., taxon or gene) level. In microbiome studies, the clustering of microbial taxa is more common; that is, to identify patterns in microorganisms that evolve similarly over time. For microbial community analyses, clustering proves beneficial in identifying natural groupings or partitions within samples [98]. The inclusion of time in longitudinal microbiome data enables us to discern patterns in microorganisms that exhibit similar evolutionary trajectories over time. Coenen et al. [50] reviewed the application of time series clustering methods to microbiome datasets, highlighting the importance of meticulous consideration of metrics and normalization methods in dealing with the high-dimensional and over-dispersed nature of microbiome data. Gibson and Gerber [20] proposed a novel dynamical systems model termed “interaction modules”, which are clusters of latent variables with a redundant interaction structure. This reduces the expected number of interaction coefficients significantly. The model is fully Bayesian, propagating measurement and latent state uncertainty throughout the model and incorporates a temporally varying auxiliary variable technique to facilitate efficient inference by relaxing the non-negativity constraint. Additionally, wavelet clustering analysis proposed by Benincà et al. [83] is a novel approach designed to examine spectral characteristics and temporal dynamics of microbial communities. Unlike traditional correlation-based methods that might offer limited or biased insights, wavelet clustering uses a periodic function, the mother-wavelet, to assess and determine statistical significance in periodicities. A distance matrix is then computed from the wavelet power spectra, incorporating leading patterns and singular vectors from matrix decomposition analysis. This method allows for the extraction of more nuanced information on dependencies within microbial communities and can reveal community structures that remain obscured in correlation-based methods.

### 6.3. Dynamic Interaction Network Analysis

Microbiome interaction networks infer the complex relationships between microbiome community members and can be used to optimize the search for potential intervention (e.g., therapeutic) targets. While these networks are informative, constructing them can be challenging, as it often involves solving NP-hard problems, requiring significant computational resources and sophisticated algorithms. The complexity arises from the need to model intricate relationships between microbial community members and the high data dimensionality, which can lead to overfitting and computational challenges.

Inferences can emanate from parametric models, wherein between-taxa interactions are characterized via parameter estimation. A prominent example of such an approach is the application of the gLV model to incorporate temporal dynamics, as employed by MDSINE [74]. The gLV model naturally considers interactions within its framework, allowing for a more intuitive representation of microbial community relationships. Specifically, in Equation (2), the parameter $\beta_{lj}$ symbolizes the microbial interactions between taxon *l* and *j*, serving as the foundation for constructing the interaction network. This method has been further developed and implemented by other groups including Jover et al. [84], who utilized the gLV model to develop a phage–bacteria infection network.

Chen et al. [72] constructed an interaction network based on the above-mentioned state space model. These investigators used a high-dimensional linear State Space Model (SSM) coupled with an expectation-regularization-maximization (ERM) algorithm. By employing the adaptive LASSO-based variable selection method, the SSM allowed for the construction of a dynamic microbial interaction network (MIN), preserving the sparsity property of MINs. Unlike similar models, the SSMs equipped with the ERM algorithm considered systematic error and measurement error separately, enhancing the accuracy of the dynamic network.

Bayesian network-based methods have emerged as powerful tools for modeling the gut microbial ecosystem among other niches, and certain represent a growing trend in the field of longitudinal microbiome data analyses. Utilizing tools such as conditional Gaussian Bayesian networks (CGBayesNets) [99], investigators have employed simplified two-stage dynamic Bayesian networks (TS-DBN) that model the relationships between consecutive time points, deliberately excluding transitions within individual time points. This approach has been further refined through the integration of advanced techniques such as spline estimation and DTW, which enable the alignment of microbial relative abundance data across time. By leveraging these aligned time series, investigators can then construct more accurate dynamic Bayesian networks (DBNs), as evidenced by their improved prediction performance in longitudinal microbiome studies. The field continues to evolve with the development of innovative methods designed to infer complex causal relationships between microbial taxa, clinical endpoints (e.g., disease statuses), and person-level characteristics (e.g., demographic factors). A notable example of advancements in this area is the computational pipeline PALM (pipeline for the analysis of longitudinal multi-omics data) proposed by Ruiz-Perez et al. [85]. PALM represents a significant novel contribution, as it aligns multi-omics data and employs DBNs to create a unified model. This approach effectively navigates the challenges of differing sampling and progression rates, employs a biologically inspired multi-omics framework, and manages the complexity of the large number of features and parameters within the DBNs.

Granger causality has emerged as a widely utilized model for constructing interaction networks within the field of microbiome research. This statistical approach investigates postulated causal associations between two entities, for example, between taxon ‘A’ and taxon ‘B’, by examining how past values of one can predict the future values of the other. Specifically, if the past values of taxon A contain information about the future values of taxon B that would not be available otherwise, A is said to ‘Granger cause’ B. Several studies have applied Granger causality to construct microbial causality networks. Ai et al. [86] utilized this method to analyze data from the San Pedro Ocean Time-Series (SPOT) [100] and the Plymouth Marine Lab (PML) [101], while Mainali et al. [87] employed it to build an interaction network for the human microbiome using data from Caporaso et al. [40].

### 6.4. Classification of Participants in Longitudinal Microbiome Studies

Participant classification commonly revolves around the prediction of host traits such as health and disease statuses, treatment responses, etc. [102]. Traditional statistical methods have been foundational for classification analyses in cross-sectional datasets, as extensively reviewed by Zhou and Gallins [103]. However, classifying longitudinal microbiome data is substantially more complex because it requires modeling both temporal patterns and species interactions with other charateristics such as health and disease statuses. This complexity often exceeds what traditional methods can handle.

One approach to this challenge is to first delineate the interaction pattern explicitly, as previously discussed, and then utilize this pattern for prediction. Alternatively, deep learning algorithms have emerged as another potential solution, owing to their exceptional representational power. These algorithms, capable of learning hierarchical data representations and modeling intricate relationships between variables, are particularly well-suited for the classification of microbiome time series data. Recent advances include the development of specialized deep learning models that leverage techniques such as convolutional neural networks (CNN) [104] and long short-term memory (LSTM) [105] networks to process sequential data and capture temporal dependencies.

Recurrent neural networks (RNNs) [106], designed specifically for sequential data, play a vital role in this context. RNNs possess the ability to “remember” information from previous time points, integrating it into the processing of the subsequent time point. Consequently, prediction results at a given time *t* are determined by both the input information at *t* and the preceding information from 1 to t−1 .

However, conventional RNN models are often plagued by the vanishing gradient problem, particularly when dealing with a large number of time points. This issue can render the gradient of certain parameters with respect to the loss function too minuscule to facilitate effective learning. To overcome this limitation, LSTM networks were introduced. As a specialized variant of RNNs, LSTM networks selectively retain crucial information from previous time points, alleviating the vanishing gradient problem and enabling the effective modeling of long-term dependencies in longitudinal data. This makes LSTM networks a valuable tool for modeling microbiome time series data.

For practical applications, Metwally et al. [88] employed an LSTM model to explore the relationship between allergy development and time patterns in the infant gut microbiome. These investigators innovatively utilized an autoencoder to extract taxonomic information at each time point into a single latent feature, followed by an LSTM model to link and aggregate these features, uncovering relationships between response variables and aggregated microbiome information. Sharma and Xu [89] introduced phyLoSTM, a novel method for classifying microbiome time series data. Unlike previous studies that used an autoencoder, phyLoSTM employed a CNN to extract information at each time point. However, it retained the use of an LSTM model to aggregate information across different time points, identifying the relationship between response variables and temporal patterns in the data.

### 6.5. Other Microbiome Time-Series Related Analyses

There are also other valuable microbiome-related analytical approaches that exist and are worth considering. One such example is the identification of stable states, which is a crucial aspect of understanding the behavior of microbial ecosystems. Determining the stable states of a microbiome system can reveal valuable information about the dynamics of the system and its level of resilience, as well as potential avenues for intervention and manipulation. Stein et al. [75] define a stable state as a condition in which the abundance of each species is either zero or its growth rate is zero.

Another interesting research question is related to the identification of keystone species in a microbial system. Keystone species are those that have a disproportionate influence on the functioning of an ecosystem, and their identification can provide insights into the potential drivers of change within the system. Fisher et al. [107] also paid attention to the inference of keystoneness in microbiome data. They defined microorganisms that have a higher number of interactions than others as the “dominant microbiome”. Bucci et al. [74] measured keystoneness in a different way. The measure departs from a community composition that allows the largest number of taxa to stably coexist, and then removes each taxon from the community in turn. The taxa are then ranked based on the magnitude of the Euclidean distances obtained as a consequence of their removal.

## 7. Conclusions and Discussion

Here, we comprehensively reviewed several key aspects of longitudinal microbiome data, with a focus on a few very recent developments specifically for longitudinal microbiome data. In addition to the standard statistical methods [12], we also include the statistical model gLV for interaction module [76] and a deep learning-based method phyLoSTM for prediction [89].

As microbiome research advances, an increasing number of techniques tailored for the management of complex data structures emerge. All these methods, including statistical and deep learning methods, have strengths and limitations. Statistical methods are usually easy to interpret and are computationally efficient due to the relatively small number of parameters. However, they rely on several assumptions, which can lead to inaccurate results when violated. Meanwhile, deep learning methods, which have seen rapid growth and development in recent years, are becoming a popular choice among investigators working with microbiome data. Deep learning methods are highly flexible and can model complex non-linear relationships, especially in large datasets. Furthermore, deep learning models can be integrated with other types of data, such as person-level characteristics and clinical health and disease endpoints. However, deep learning methods are usually regarded as a black box with inexplicable inner workings [108], creating challenges at the stage of results interpretation. Because there are highly numerous parameters in deep learning models, they almost always require voluminous, high-quality, and correctly-labeled data to retain reliability [108] and may be computationally demanding.

Several directions exist for improving model performance for longitudinal microbiome data analyses. One approach is to develop more comprehensive statistical models that can account for the complex features of microbiome data structures. However, more comprehensive models will inevitably incur additional parameters, which require more high-quality data and additional computational resources to fit the model. As for deep learning models, one should anticipate development of more tailored approaches for the complex microbiome data structures instead of adapting models from other fields that may not perform optimally. Furthermore, the combination of statistical methods and deep learning methods may be a promising research avenue to explore. Until now, there are have been few attempts to combine these methods for microbiome data analysis. Because deep learning models, while high-performing, are often considered as black boxes, identifying ways to use them in conjunction with statistical methods could offer gains in interpretability, and is an open area for future methods development.

Methodological developments seeking to improve the performance of models for longitudinal microbiome data analysis requires access to high-quality datasets, and these are currently scant. Both statistical methods and deep learning approaches benefit from larger and more comprehensive datasets. Meanwhile, combining different types of biological information (e.g., multi-omics data) could also help advance insights gained from microbiome data analyses; e.g., one can co-analyze the dynamics of microbial abundance and metabolism [109]. Multi-omics data may help illuminate, better characterize, and even predict shifts in community function over time [110]. Looking into the future, we anticipate that larger sample sizes of longitudinal microbiome data will catalyze the development and implementation of advanced methods involving statistical methods and deep learning methods to leverage biological information and predict clinical outcomes.

Due to the limited number of available large longitudinal microbiome data analysis and the needs of new computational methods, we may leave comparison of these computational methods using real data for future publications. Unlike most review papers that summarize and compare well-established methods in a field, we hope this paper provides timely insights while this longitudinal microbiome field is still in the relative early stage. 

## Figures and Tables

**Table 1:** Summary of normalization methods in microbiome data analysis.

Method	Category	Equation	Brief Description	Use Cases & Limitations
ALDEx2 [52]	Log-ratio	$\ln\left(Y_{l}\right)-\ln\left(Ref\right)$	Converts observed abundances to log-ratios with a reference taxon.	Good for compositional data; dependent on choice of reference taxon.
CLR (Centered Log-Ratio) [53]	Log-ratio	$\ln\left(Y_{l}\right)-\frac{1}{n}\sum_{l=1}^{L}\ln\left(Y_{l}\right)$	Subtracts log of mean abundance from log of individual taxon abundance.	Popular in microbiome analysis; assumes constant sum across samples.
Z-score (Standard Score)	Scaling	$\frac{Y_{l}-\mu_{Y}}{\sigma_{Y}}$	Normalizes data to mean 0 and standard deviation 1.	Useful for Gaussian distribution assumptions; sensitive to outliers.
MED (Median Normalization) [53]	Scaling	$\frac{Y_{l}}{Median(Y)}$	Uses median intensity within a sample for scaling.	Robust against outliers; simple and effective.
UQ (Upper Quartile)	Scaling	$\frac{Y_{l}}{Q3(Y)}$	Scales based on the 75th percentile (Q3) intensity.	Useful for range variation; does not adjust for compositional data.
TMM (Trimmed Mean of M-values) [55]	Scaling	$\frac{Y_{l}}{TrimmedMean(Y)}$	Adjusts gene expression ratios, trimming extreme values for mean calculation.	Good for RNA-Seq data; may not be ideal for all microbiome data types.
TSS (Total Sum Scaling)	Scaling	$\frac{Y_{l}}{\sum_{i=1}^{N}Y_{i}}\times T$	Standardizes total feature intensities to a fixed value T across samples.	Ideal for studies focusing on total microbial load or gene expression levels; can be skewed by high-abundance features.
CSS (Cumulative Sum Scaling) [56]	Scaling	$\frac{Y_{l}}{\sum_{i=1}^{N}Y_{i}}\times C$	Scales data to a consistent cumulative sum (C) based on the dataset characteristics. Adjusts for varying signal intensities/sample depths.	Suitable for datasets with varying sequencing depths; preserves relative differences in lower abundance features. Doesn’t account for compositional nature.

**Table 2:** Summary of statistical models in longitudinal microbiome data analysis.

Models	Brief Description	Examples
Mixed effect models	Models handling population-level trends (fixed effects) and individual variations (random effects)	Bokulich et al. [70], Chen et al. [25], Zhang et al. [26], Zhang et al. [27]
ARIMA models	Models combining autoregressive (AR) terms, differencing (I), and moving average (MA) terms	Benjamin et al. [71]
State space models	Probabilistic graphical models that describe the probabilistic dependence between latent state variables and observed measurements	Chen et al. [72]
Principal trend analysis models	Models that are used to identify and assess the main trends in a dataset over time or across different conditions	Wang et al. [73]
Generalized Lotka–Volterra models	Models expressed like Equations allowing dynamic interaction among taxa (2)	Bucci et al. [74], Stein et al. [75], Gibson et al. [20], Shaw et al. [31], Kuntal et al. [76]
Bayesian models	Models using Bayesian methods for parameter inference	Äijö et al. [48], Silverman et al. [47], Joseph et al. [49]
